# How does the multifaceted plant hormone salicylic acid combat disease in plants and are similar mechanisms utilized in humans?

**DOI:** 10.1186/s12915-017-0364-8

**Published:** 2017-03-23

**Authors:** D’Maris Amick Dempsey, Daniel F. Klessig

**Affiliations:** Boyce Thompson Institute, 533 Tower Rd, Ithaca, NY 14853 USA

## Abstract

Salicylic acid (SA) is an important plant hormone that regulates many aspects of plant growth and development, as well as resistance to (a)biotic stress. Efforts to identify SA effector proteins have revealed that SA binds to and alters the activity of multiple plant proteins—this represents a shift from the paradigm that hormones mediate their functions via one or a few receptors. SA and its derivatives also have multiple targets in animals; some of these proteins, like their plant counterparts, are associated with pathological processes. Together, these findings suggest that SA exerts its defense-associated effects in both kingdoms via a large number of targets.

## What is the plant hormone salicylic acid?

Salicylic acid (SA; 2-hydroxybenzoic acid) is one of many phenolic compounds (defined as compounds containing a benzene ring bearing one or more hydroxyl groups) that are synthesized by plants. Despite the diversity and ubiquity of plant phenolics, these compounds were traditionally assumed to be rather unimportant, secondary metabolites. However, phenolics were subsequently shown to be involved in many important processes, including lignin and pigment biosynthesis, allelopathy, and the regulation of responses to abiotic and biotic stresses [1]. SA, for example, is a critical hormone that plays direct or indirect roles in regulating many aspects of plant growth and development, as well as thermogenesis and disease resistance [2]. Beyond its functions in plants, SA and its acetylated derivate (commonly known as aspirin) are important pharmacological agents for humans. SA is commonly used to treat warts, acne, and psoriasis, while aspirin is one of the most widely used medications in the world; its uses include treating pain, fever, swelling, and inflammation, as well as reducing the risk of heart attack, stroke, and certain cancers [3–5].

## How was SA’s status as a hormone discovered?

The first evidence that SA is a plant hormone came from studies of voodoo lily (*Sauromatum guttatum* Schott) [6]. During blooming, the voodoo lily inflorescence exhibits two episodes of thermogenesis (heat production). These events increase the surface temperature of the inflorescence by 12 and 10 °C and are thought to volatilize compounds that attract/stimulate insect pollinators. Internal SA levels increased ~100-fold prior to each episode [7]. Externally supplied SA and two closely related analogs also induced thermogenesis, whereas 31 other SA analogs did not. SA induces thermogenesis primarily by stimulating the mitochondrial alternative respiratory pathway [8]. This pathway, unlike the cytochrome respiratory pathway, generates ATP at just one step and releases the remaining energy from electron flow as heat. Interestingly, SA treatment also induces the expression of alternative oxidase and/or the alternative respiratory pathway in non-thermogenic plant species [9, 10].

The year after SA’s function in thermogenesis was elucidated, its role as a defense signaling hormone was documented (see below). SA also has long been proposed as a signal for flowering [11]. Consistent with this possibility, the early flowering phenotype observed in several *Arabidopsis* mutants correlates with, and is dependent on, elevated SA levels [12–15]. In addition, flowering was delayed in one study of SA-deficient *Arabidopsis* [16], although others failed to observe this phenomenon [17, 18]. Interestingly, several proteins that regulate both flowering and resistance signaling have been identified [12, 13, 15, 17–21]. While this finding suggests an interconnection between flowering and disease resistance [22], it is clearly complex: some of these proteins regulate both resistance and flowering in an SA-dependent manner, whereas several others regulate resistance via an SA-dependent pathway but positively or negatively regulate flowering via an SA-independent mechanism.

## How do plants resist pathogen infection?

Although plants lack the circulating immune cells found in vertebrates, they do possess an innate immune system that detects and limits pathogen colonization [23–25]. One branch of this system uses pattern recognition receptors (PRRs) on the plant cell surface to survey for molecules containing characteristic patterns that are unique to, and broadly conserved in, microbes. Detection of these pathogen-/microbe-associated molecular patterns (PAMPs/MAMPs) leads to activation of pattern-triggered immunity (PTI). In many cases, PTI prevents further pathogen colonization. However, some pathogens have evolved effector proteins that suppress PTI. These pathogens are combatted via effector-triggered immunity (ETI), which comprises the other branch of the innate immune system. ETI is activated when plant-encoded resistance (R) proteins, which are generally located within the plant cell, directly or indirectly recognize their cognate pathogen-encoded effectors. Both PTI and ETI are associated with the activation of defenses in the inoculated tissue, including the generation of reactive oxygen species (ROS), increases in intracellular Ca^2+^ concentrations, activation of mitogen-activated protein kinases (MAPKs), increased expression of various defense-associated genes, synthesis of antimicrobial compounds and accumulation of SA [26, 27]. Generally, ETI induces these defenses more rapidly and intensely than PTI. ETI also is usually associated with necrotic lesion formation, which may help restrict pathogen movement from the infection site. Subsequent to these events, ETI and PTI can induce immune responses in the uninoculated (systemic) portions of the plant, including a long-lasting, broad-spectrum resistance called systemic acquired resistance (SAR) [2, 23, 28].

## How was SA’s role as a hormone signaling disease resistance discovered?

Since the late 1970s, it was known that applying SA to tobacco plants induces defense gene expression and enhances resistance to viral infection [29]. However, SA’s role as an internal signal for disease resistance was not demonstrated until 1990, when rises in SA levels were detected prior to the development of local and/or systemic disease resistance in tobacco and cucumber [30, 31]. Analyses of tobacco and *Arabidopsis* unable to accumulate SA (due to various mutations or expression of SA-degrading enzymes) confirmed that SA is required for PTI, ETI, and SAR [2]. Grafting studies using SA-deficient or wild-type (wt) tobacco further indicated that while SA accumulation is required in the uninfected leaves for SAR development, SA is not the mobile SAR-inducing signal that travels from the inoculated to systemic leaves [32, 33]. SA’s role as a defense signal has been extended to many plant species. However, there are conflicting reports regarding its role in some monocots [2], as well as in plants that constitutively accumulate high levels of SA (such as potato and rice); its role in some plant species also appears to vary depending on the pathogen [34–37].

It should be noted that SA is one of several plant hormones involved in signaling defenses against microbial pathogens [38, 39]. The SA-mediated defense signaling pathway is activated following infection by biotrophic pathogens, which require living host tissue. By contrast, attack by necrotrophic pathogens, which feed on dead tissue, induces a distinct defense pathway that is regulated by the plant hormones jasmonic acid (JA) and ethylene. The SA- and JA/ethylene-mediated defense pathways undergo extensive cross-talk; their interactions are generally antagonistic.

## How do plants synthesize SA?

**Fig. 1.**
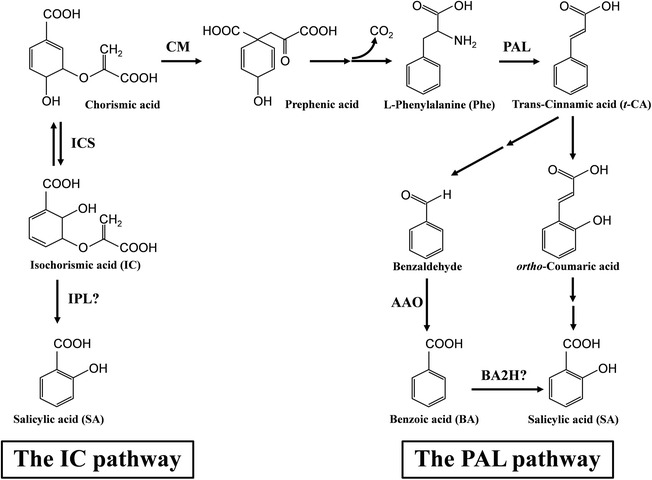
Plants have two pathways for SA production, the isochorismate (*IC*) pathway and the phenylalanine ammonia-lyase (*PAL*) pathway. Enzymes involved in SA biosynthesis are abbreviated as follows: *AAO* aldehyde oxidase, *BA2H* benzoic acid 2-hydroxylase, *CM* chorismate mutase, *ICS* isochorismate synthase, *IPL* isochorismate pyruvate lyase, and *PAL* phenylalanine ammonia-lyase. *Question marks* indicate that the enzyme responsible for the indicated conversion has not yet been definitively identified

Plants utilize the isochorismate (IC) and the phenylalanine ammonia-lyase (PAL) pathways to synthesize SA, as well as many other important compounds (Fig. 1; for more information, see [40]). Although neither route for SA biosynthesis is completely understood, both are known to require the primary metabolite chorismate [40–42]. In the PAL pathway, PAL converts phenylalanine (Phe) to *trans*-cinnamic acid (*t*-CA). Depending on the plant species, *t*-CA is converted to SA via the intermediates *ortho*-coumaric acid or benzoic acid (BA). The conversion of BA to SA presumably occurs via BA 2-hydroxylase. The IC pathway was identified based on the hypothesis that plants synthesize SA via a pathway analogous to that of some bacteria [43]. Indeed, genes encoding isochorismate synthase (ICS), which converts chorismate to isochorismate, have been identified in many plant species. However, no plant gene corresponding to bacterial isochorismate pyruvate lyase, which converts isochorismate to SA and pyruvate, has been identified. Following its synthesis, *Arabidopsis* ICS1 is imported to the chloroplast stroma, where SA synthesis occurs [44].

Demonstrating the IC pathway’s importance, *Arabidopsis ics1* mutants (also designated *SID2* or *EDS16*) accumulate 90–95% less pathogen-induced SA and display decreased disease resistance [43, 45, 46]. Similarly, pathogen-induced SA was suppressed in *ICS*-silenced tomato and *Nicotiana benthamiana* [47, 48]. Furthermore, diverse pathogens secrete chorismate- or isochorismate-metabolizing enzymes that promote virulence by suppressing plant SA accumulation [49, 50]. Since *Arabidopsis* mutants defective for both *ICS* genes lack ICS activity but accumulate low SA levels [51], an alternative, presumably PAL-dependent, pathway must also be functional. Indeed, *PAL*-deficient *Arabidopsis*, tobacco, and pepper exhibited reduced pathogen-induced SA accumulation and disease resistance, whereas *Arabidopsis* overexpressing *CaPAL1* from pepper displayed increased pathogen-induced SA accumulation and resistance [33, 52, 53]. It should be noted, however, that pathogen-induced SA accumulation still occurs in PAL-suppressed plants, while it is largely abrogated in ICS-deficient plants. Whether PAL suppression affects SA accumulation by impacting the phenylpropanoid pathway or via an indirect effect on SA precursors, such as chorismate, is unclear [52].

## How is SA synthesis regulated?

Analyses of *Arabidopsis* mutants have identified several proteins required for pathogen-induced SA accumulation, including enhanced disease susceptibility 1 (EDS1) and phytoalexin deficient 4 (PAD4), which are lipase-like proteins, and non-race-specific disease resistance 1 (NDR1), a glycophosphatidyl inositol-anchored plasma membrane protein [2, 40]. EDS1 forms cytoplasmic- and nuclear-localized complexes with various resistance-associated proteins [54, 55], including PAD4 [56]. During ETI, the nuclear pool of EDS1 increases rapidly; this precedes/coincides with *ICS1*, *PAD4*, and *EDS1* upregulation [57]. While EDS1, PAD4, and SA comprise a positive feedback loop [56], the mechanism through which EDS1/PAD4 influence *ICS1* expression is unknown.

Several transcription factors (TFs) that positively regulate *ICS1* expression have been identified [26, 58], including calmodulin-binding protein 60 g (CBP60g) and its homolog SAR deficient 1 (SARD1) [59, 60], WRKY28 [61], teosinte branched1/cycloidea/PCF 8 and 9 (TCP8 and 9) [62], NTM-like 9 (NTL9) [63], and CCA1 hiking expedition (CHE; also designated TCP21) [63, 64]. Ca^2+^ is implicated in CBP60g [65] and WRKY28 [66] activation, suggesting a mechanism linking pathogen-induced Ca^2+^ influxes with SA biosynthesis [26]. NTL9 is preferentially expressed in guard cells and is required for PTI-associated stomatal immunity, and CHE, a component of the circadian clock, regulates both circadian oscillation of *ICS1* expression and its systemic induction during SAR. Interestingly, TCP8 interacts with several other TCPs and *ICS1*-associated TFs [62]; these interactions could confer nuanced *ICS1* expression and also form regulatory nodes between different signaling pathways [63]. Transcriptional repressors of *ICS1* include ethylene insensitive 3 (EIN3), EIN-like 1 (EIL1), and three NAC TFs, ANAC019, ANAC055, and ANAC072 [58]. Since these TFs also regulate JA and ethylene signaling, they may mediate cross-talk between these pathways.

Interestingly, a chloroplast-localized, calcium-sensing receptor (CAS) is required for full activation of various ETI-/PTI-induced defenses, including SA accumulation, stromal Ca^2+^ fluxes, stomatal closure, nuclear-encoded defense gene expression, and pathogen resistance [67]. Although the mechanism(s) through which CAS exerts its effects is unclear, these findings suggest that retrograde signals from chloroplasts also regulate SA biosynthesis and immunity [27].

## How are cytosolic SA levels regulated?

**Fig. 2.**
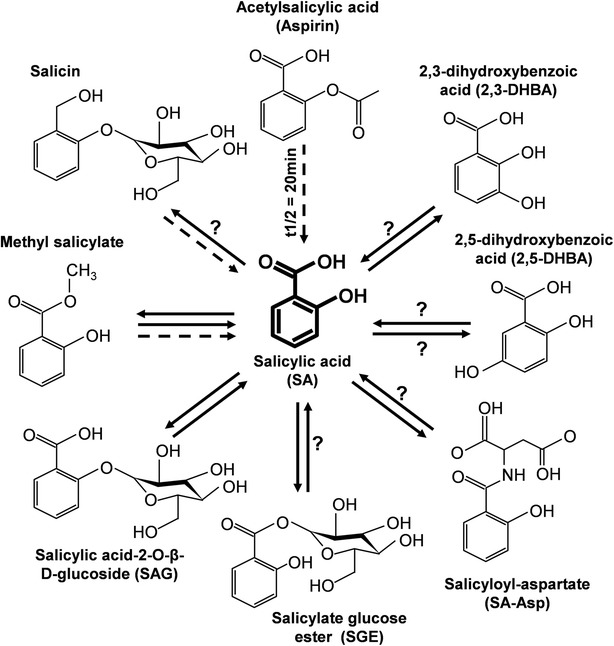
SA undergoes many modifications in plants. The level of biologically active SA in the cytoplasm is regulated by an array of modifying enzymes that convert it to biologically inactive derivatives (reviewed in [40]). Shown here are the structures of SA and its derivatives; their interconversions are indicated by *solid arrows*. The prodrugs salicin, which is a natural salicylate, and aspirin, a synthetic SA derivative, are also depicted; their conversion to SA in animals is indicated by *broken arrows*. Methyl salicylate (*MeSA*) is both a transported form of SA *in planta* and a prodrug in humans; thus, the *in planta* conversions are depicted by *solid arrows*, while the human conversion of the prodrug is represented as a *dashed line. Question marks* indicate either that the reaction steps have not yet been identified or that the enzyme responsible for the indicated conversion has not yet been definitively identified. In animals, aspirin is rapidly converted to SA with a half-life (*t1/2*) of approximately 20 min

After synthesis in the chloroplast, SA is transported to the cytosol where it signals immune responses. In *Arabidopsis*, SA export is likely mediated by EDS5 (also designated SID1), a chloroplast envelope-localized member of the multidrug and toxin (MATE) transporter family [68, 69]. Once in the cytoplasm, SA can undergo various modifications that generally render it inactive (Fig. 2) [40, 41, 58]. These modifications are thought to help regulate the level of biologically active SA in the cytoplasm, provide a rapidly accessible source of SA and/or facilitate SA transport throughout the plant. Maintaining low cytoplasmic SA levels except during immune signaling is critical, as constitutive activation of immunity reduces plant fitness by shunting both energy and resources away from growth and reproductive processes [70]. Glucosylation of SA at its hydroxyl group generates SA 2-*O*-β-D-glucoside (SAG), while glucosylation at its carboxyl group produces salicylate glucose ester (SGE). SAG is transported to the vacuole, where it serves as a non-toxic storage form that can be hydrolyzed to release free SA following pathogen attack. Methylation of SA generates methyl SA (MeSA). MeSA is a phloem-mobile SAR signal that travels from the infected leaf to the systemic tissues, where it activates resistance following its conversion back to SA [71]. Pathogen-infected *Arabidopsis* also accumulate 2,3-dihydroxybenzoic acid (2,3-DHBA) and, to a lesser extent, 2,5-DHBA [72]. Conversion of SA to 2,3-DHBA is mediated by SA 3-hydroxylase (S3H; also termed DLOL1) [73, 74]. Since *s3h* mutants accumulate high levels of SA, S3H-mediated hydroxylation may be a critical mechanism for preventing SA over-accumulation [73]. Formation of SA–amino acid conjugates is yet another modification strategy [40]. Salicyloyl-aspartate (SA-Asp) has been identified in plants; it is presumably synthesized by GH3.5 (also termed WES1), a member of the GH3 acyl adenylase family [75]. Another GH3 family member, GH3.12 (also termed PBS3, GDG1, and WIN3), conjugates 4-substituted benzoates to Glu [76]. Since *gh3.12* mutants display reduced pathogen-induced SA/SAG accumulation and disease resistance, and these phenotypes were rescued by SA, GH3.12 was predicted to function upstream of SA synthesis/accumulation [40]. Given that stress-treated *gh3.12* mutants accumulate elevated levels of SA-Asp, GH3.12 may increase cytosolic SA levels by repressing SA-Asp synthesis.

## What plant processes does SA affect?

In *Arabidopsis*, the basal level of total SA (consisting of SA and SAG/SGE) in leaves ranges from 0.22–5 μg/g fresh weight; in the Solanaceous species tobacco and potato it is <0.4 μg/g fresh weight and <17 μg/g fresh weight, respectively [77]. Given the wide range in basal SA levels between (and even within) plant species, it is perhaps not surprising that conflicting reports have been published concerning the effect of exogenously supplied SA on various plant processes. Despite this caveat, exogenous SA has been shown to affect resistance to biotic (pathogen-associated) stress and tolerance to many abiotic stresses (drought, chilling, heat, heavy metal, UV radiation, and salinity/osmotic stress), as well as multiple aspects of plant growth and development, including seed germination, vegetative growth, flowering, fruit yield, senescence, thermogenesis, stomatal closure, root initiation/growth, photosynthesis, respiration, glycolysis, Krebs cycle, and the alternative respiratory pathway [42, 78–81]. Some of these processes are induced by SA in a concentration-dependent manner, as they are activated by treatment with a low dose of SA and inhibited by a high dose. This phenomenon is likely linked to SA’s role in regulating cellular redox status, as low concentrations of SA induce low-level accumulation of ROS, which serve as secondary signals to activate biological processes. By contrast, high concentrations of exogenous SA stimulate the accumulation of high levels of ROS, which cause oxidative stress and cell death [2]. Currently, the mechanisms through which SA regulates these non-immune plant processes are not well understood, but they do appear to involve the coordinated effect of SA and other plant hormones.

## How does SA work—a few receptors or multiple targets?

It is generally assumed that plant and animal hormones signal downstream responses by binding to one or a small number of receptors. Whether this paradigm can be extended to SA is currently unclear. In *Arabidopsis*, members of the non-expressor of pathogenesis-related genes (NPR) protein family were proposed to be SA receptors [82–84]. SA binding to NPR1 (also designated NIM1 and SAI1) may promote immune signaling by relieving sequestration of NPR1’s trans-activating domain (see below) [83]. By contrast, NPR3 and NPR4 appear to be negative regulators of immunity [82]. Identification of these putative SA receptors represents progress. However, several lines of evidence argue that additional SA-binding proteins (SABPs) mediate SA’s functions. First, some immune responses are activated via an SA-dependent but NPR1-independent pathway(s). Second, there is little evidence that non-immune processes affected by SA are NPR1-dependent. Indeed, nearly 30 SABPs have been identified; these proteins exhibit a range of affinities for SA and their activities are altered by SA binding [77, 85]. Given that SA levels can vary dramatically between plant species and also within a plant, depending on its developmental stage, the tissue type, subcellular compartment, and exposure to (a)biotic stress, it is possible that SA signals its many effects by interacting with different SABPs, depending on their location and SA affinity [77]. This hypothesis represents a paradigm shift for how at least some hormones function. We recently proposed that proteins whose activity is altered following hormone/ligand binding be termed “targets”, and only a subset of targets that meet additional criteria be designated “receptors”. Determining which criteria must be met to qualify an SABP as an SA receptor will be complicated since i) SA binding alters the activity of many SABPs and ii) some SABPs exhibit comparably high affinity as the proposed receptors NPR1 and NPR4, while others display a low affinity similar to NPR3.

## What is the best-defined SA signaling pathway?

SA signals defense responses via both NPR1-dependent and NPR1-independent pathways; of these, the NPR1-dependent pathway is the best understood. *Arabidopsis npr1* mutants fail to activate immunity following SA treatment or pathogen infection, indicating NPR1’s critical importance for SA signaling [41, 58]. NPR1 contains two domains mediating protein–protein interactions: a BTB/POZ and an ankyrin repeat domain, as well as a C-terminal trans-activating domain and nuclear localization sequence [86]. In uninfected plants, NPR1 is predominantly sequestered in the cytosol as oligomers. After infection, SA-induced changes in the cellular redox state reduce NPR1 to monomers that are transported to the nucleus. There, NPR1 co-activates transcription of immune-associated genes [87]. For the defense marker gene *pathogenesis-related-1* (*PR-1*), NPR1 directly interacts with members of the TGA transcription factor family [2, 26, 88]. *PR-1* expression may be further regulated by interactions between NPR1 and members of the non-inducible immunity 1 (NIM)-interacting (NIMIN) family, and by proteins proposed to function downstream of NPR1 [26, 88]. NPR1 also upregulates the expression of WRKY TFs, which regulate many immunity-associated genes [89]. WRKY binding sites are located in the *NPR1* and *ICS1* promoters, suggesting the presence of a feedback loop that fine-tunes SA signaling [26].

To prevent spurious/inappropriate induction of immune responses, NPR1 activity is tightly regulated. Besides nuclear translocation, SA binding at two cysteine residues (Cys521 and Cys529) may regulate NPR1 activity by inducing a conformational change that releases the trans-activating domain from the inhibitory BTB/POZ domain [83]. How this mechanism applies to plant species whose NPR1 homologs lack Cys521 and Cys529 is unclear [86, 90]. NPR1 activity also is regulated via proteasome-mediated degradation [91]. This process may be mediated by NPR3 and NPR4, which are adaptors for Cullin 3 ubiquitin E3 ligase [82]. NPR4 is proposed to maintain low NPR1 levels in uninfected cells. Following infection, SA disrupts the NPR1–NPR4 interaction, allowing NPR1 to accumulate and defense signaling to occur. In cells containing sufficiently high SA levels, NPR3 binds NPR1; this promotes NPR1 turnover, which optimizes defense activation and resets NPR1 levels [82, 91].

## How is SA research benefitting agriculture?

**Fig. 3.**
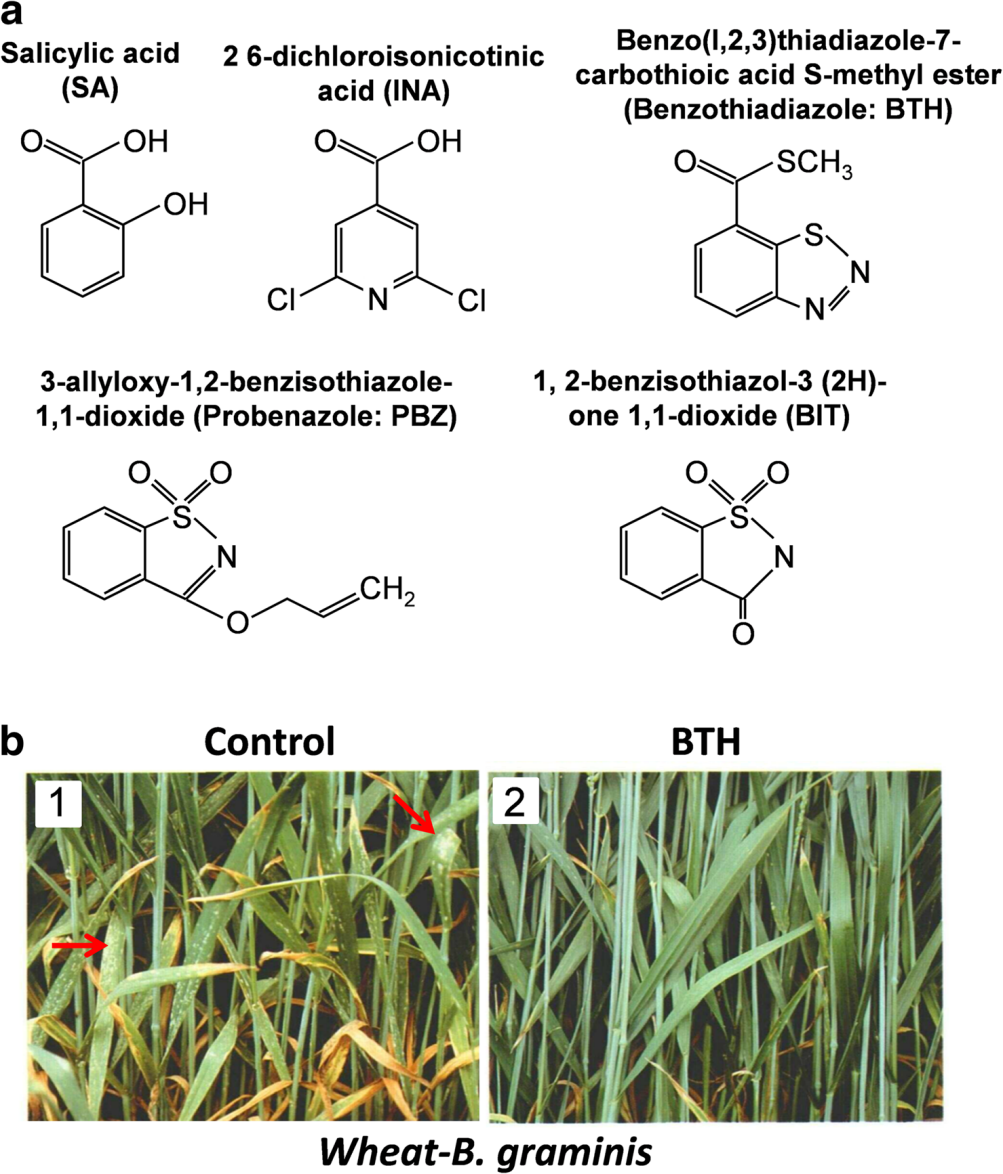
SA and several structurally and/or functionally related synthetic compounds induce SAR. **a** The chemical structures are depicted. **b** BTH treatment suppresses powdery mildew caused by *Blumeria graminis* in wheat. *Arrows* point to the *white*, powdery-like spots/blotches on the leaves of untreated wheat (*Control*), which turn *yellow* and die, whereas the leaves of BTH-treated wheat remain healthy after infection. The picture in (**b**) is copyrighted by the American Society of Plant Biologists and is reprinted from [112], and modified slightly, with the author’s and American Society of Plant Biologists’ permission

Synthetic pesticides have allowed growers to dramatically increase crop yield and quality. However, these compounds are often toxic and their overuse has led to pathogen resistance [92, 93]. An environmentally friendlier strategy for reducing crop loss involves regulating SAR [94]. SA is an effective SAR inducer, but its phytotoxicity precludes widespread use [95]. Several synthetic compounds (Fig. 3), including 2,6-dichloroisonicotinic acid (INA), benzo(1,2,3)thiadiazole-7-carbothioic acid S-methyl ester (also termed benzothiadiazole; BTH or acibenzolar-S-methyl), and probenazole (PBZ) and its active metabolite 1,2-benzisothiazol-3(2H)-one 1,1-dioxide (BIT), induce defense gene expression and SAR to a similar range of pathogens as SA [96, 97]. PBZ and BIT activate SAR by triggering the SA signaling pathway upstream of SA [98], whereas INA and BTH are SA functional analogs [96, 99]. While treating plants or suspension cells with high concentrations of SA or its functional analogs directly induces defenses, low concentrations elicit little to no response. Following subsequent infection, however, defenses are activated more rapidly and/or strongly [100]. This phenomenon, termed priming, also occurs in systemic leaves of plants exhibiting SAR. Although not fully elucidated, the molecular mechanisms of priming likely involve the accumulation of transcripts and/or inactive forms of MAPKs, elevated levels of PRRs, and chromatin remodeling [95]. This latter mechanism may also promote the inheritance of defense priming.

In addition to SA, its functional analogs and PBZ/BIT, other resistance-activating compounds that work at least in part by inducing/priming SAR, have been identified [96, 97, 101]. In the field, resistance triggered by SAR inducers/primers can be broad-spectrum and long-lasting, but it is rarely complete; disease reduction ranges from 20–85% [97]. Other drawbacks growers must currently consider include i) the potential for reduced plant fitness, which may be minimized by stimulating priming versus direct induction [102], ii) variable efficacy, depending on plant cultivar and dosage, and iii) the possibility that SAR inducers will increase susceptibility to necrotrophic pathogens, due to suppression of the JA signaling pathway [96, 97]. Nevertheless, future efforts to clarify the SA signaling pathway should provide insights into how current SAR-inducing compounds can be improved, as well as identify novel pathway components that could be targeted by the next generation of agrochemicals.

## How did aspirin become a wonder drug?

Plants rich in SA and its derivatives, collectively termed salicylates, have been used for medicinal purposes for millennia. In the fourth century BC, willow leaves/bark extracts were prescribed by Hippocrates to relieve fever and the pain of childbirth [2]. Salicylate-rich plants were also used by other ancient cultures, including the Babylonians, Assyrians, and Chinese, as well as the indigenous inhabitants of the New World [42]. Although willow bark was a well-known folk remedy, its medicinal effects were first studied clinically in the mid-1700s by the Reverend Edward Stone [3]. In 1828, Johann Buchner purified the active ingredient in willow bark and named it salicin (Fig. 2). Raffaele Piria subsequently demonstrated that salicin could be split into a sugar and an aromatic compound he named SA, in reference to *Salix alba*, the Latin name for white willow [77]. Around this time, high levels of salicylates were detected in other medicinal plants, such as meadowsweet, which contains both salicin and MeSA—then called oil of wintergreen [3, 77]. These “prodrugs” are converted to SA after digestion in humans/animals [77]. Increased demand for SA in the mid-1800s led to the commercial production of synthetic SA in 1874. As SA’s price fell and its availability increased, its clinical use expanded. However, SA’s negative side effects, particularly stomach irritation, precluded long-term, high-dosage use. Research by Felix Hoffmann revealed that acetylation improved SA’s tolerability without affecting its medicinal properties [3]. Bayer and Company began synthesizing acetyl SA (ASA) in 1897 under the trade name aspirin, which was generated by combining the “a” from acetyl and “spirin” from the Latin name for meadowsweet (*Spiraea ulmaria*). Today, aspirin is one of the most widely used drugs in the world. In addition to treating fever, swelling, pain, and inflammation, aspirin is used prophylactically to reduce the risk of stroke, heart attack, and certain cancers [3–5].

## What are SA’s targets in humans?

The combined observations that i) aspirin irreversibly inhibits the cyclooxygenases COX1 and COX2, ii) these enzymes convert arachidonic acid into prostaglandins, and iii) prostaglandins induce fever, pain, inflammation, and swelling underpin the prevailing assumption that aspirin works via COX1 and COX2 inhibition [103]. However, aspirin is rapidly metabolized to SA in the human body, and SA is a weak COX1/COX2 inhibitor. Given the similarities between SA’s and aspirin’s pharmacological effects, other SA targets likely exist [77]. Indeed, 13 additional potential targets of salicylates have been identified. The SA/aspirin levels required to alter most of these putative targets’ activities are near or above toxic levels. In contrast, low micromolar SA levels in vivo alter the disease-associated activities of high mobility group box 1 (HMGB1) [104] and glyceraldehyde 3-phosphate dehydrogenase (GAPDH) [105]. HMGB1 is an abundant chromatin-associated, non-histone protein that regulates nucleoprotein complex assembly and chromatin condensation [106]. It also is a damage-associated molecular pattern (DAMP) that triggers inflammation and induces cytokine expression when released extracellularly after tissue damage or necrosis. SA binding blocks HMGB1’s pro-inflammatory activities [104]. Notably, low SA levels, while unable to inhibit COX1/COX2 enzymatic activity, suppress HMGB1-mediated induction of *Cox2* expression.

The glycolytic enzyme GAPDH is another protein whose secondary functions are affected at low SA concentrations. GAPDH likely plays a role in neurodegenerative diseases via a cell death cascade that involves GAPDH, nitric oxide, and the E3 ubiquitin ligase Siah [107]. SA binding suppresses GAPDH’s ability to translocate to the nucleus and induce cell death [105]. Additionally, GAPDH enhances hepatitis C virus replication and/or translation by binding the viral genome; SA binding to GAPDH suppresses this activity [77]. Given that SA binding also suppresses the ability of cytosolic *Arabidopsis* GAPDH to promote plant virus replication [108] and the DAMP activity of HMGB3, an *Arabidopsis* counterpart to HMGB1 [109], plants and animals share at least several disease-associated SA targets.

## Why might humans have so many SA targets?

SA is present in the urine and/or blood of humans who have not recently consumed salicylate drugs and also in a wide range of animals [110]. Since humans on a vegetarian diet contain greater levels of SA and its metabolite salicyluric acid than non-vegetarians, consuming large quantities of plants–and their associated salicylates–appears to influence SA levels to some extent [111]. However, no correlation between blood SA levels and diet was observed in herbivorous versus carnivorous animals [110]. The presence or absence of gastro-intestinal bacteria also did not affect serum SA levels. Instead, radiotracer studies suggested that SA is synthesized endogenously in humans using BA as a precursor [110]. BA, in turn, may be synthesized endogenously from phenylalanine or provided by the diet, since some fruits and vegetables contain high concentrations of BA and its salts. We previously hypothesized that the presence of low SA levels in animals, whether endogenously synthesized or provided by the diet, led to the evolution of multiple SA targets [77]. Given the myriad effects of SA’s prodrug aspirin, others have speculated that SA is a critical animal bio-regulator that plays a key role in orchestrating defense responses, analogous to its role in plants [110]. If these possibilities are confirmed, future studies will likely identify additional SA targets that are shared in plants and animals.

## What are the future directions of SA research?

Despite significant progress in elucidating the SA signaling pathway for plant disease resistance, significant knowledge gaps remain. Our understanding of the mechanism(s) through which pathogen perception is transduced by PRRs and R proteins into activation of early cellular responses and SA synthesis is fragmentary. Likewise, the enzymes involved in SA biosynthesis are not fully identified and the role of the ICS versus PAL pathways in different plant species remains unclear. The mechanisms through which *ICS1* expression is regulated, both in the nucleus and via retrograde signals from the chloroplast, also need to be determined. As for the signaling pathway downstream of SA, a crucial line of study will involve identifying SA targets/receptors and assessing their function. These analyses, combined with the development of in vivo SA detection techniques, should provide tremendous insight into how SA exerts its myriad effects. Elucidation of the SA signaling pathway should benefit agriculture by suggesting strategies for improving current SAR-inducing compounds, as well as facilitating development of novel compounds that target currently unidentified pathway components. Finally, the discovery that animals contain multiple SA targets (which may have emerged in response to low-level exposure to endogenously synthesized and/or dietary SA) opens new avenues for treating human diseases. Indeed, SA-mediated inhibition of HMGB1’s pro-inflammatory activities might explain the protective effects of low-dose aspirin use. The identification of natural and synthetic SA derivatives that suppress HMGB1’s and GAPDH’s disease-associated activities more effectively than SA suggests there is great potential for developing SA-based drugs that are even more efficacious and/or have fewer negative side effects [77]. In summary, it is highly likely that SA’s targets in animals and plants will significantly overlap. Characterizing these proteins should not only clarify how SA induces its effects in both kingdoms, but also suggest novel strategies for controlling pathological processes.

## References

[1] Métraux JP, Raskin I, Chet I (1993). Role of phenolics in plant disease resistance. Biotechnology in plant disease control.

[2] Vlot AC, Dempsey DA, Klessig DF (2009). Salicylic acid, a multifaceted hormone to combat disease. Annu Rev Phytopathol.

[3] Weissmann G (1991). Aspirin. Sci Am.

[4] Antithrombotic Trialists’ (ATT) Collaboration (2009). Aspirin in the primary and secondary prevention of vascular disease: collaborative meta-analysis of individual participant data from randomised trials. Lancet.

[5] Cuzick J, Thorat MA, Bosetti C, Brown PH, Burn J, Cook NR, Ford LG, Jacobs EJ, Jankowski JA, LaVecchia C, Law M, Meyskens F, Rothwell PM, Senn HJ, Umar A (2015). Estimates of benefits and harms of prophylactic use of aspirin in the general population. Ann Oncol.

[6] Raskin I (1992). Role of salicylic acid in plants. Annu Rev Plant Physiol.

[7] Raskin I, Turner IM, Melander WR (1989). Regulation of heat production in the inflorescences of an *Arum* lily by endogenous salicylic acid. Proc Natl Acad Sci U S A.

[8] Rhoads DM, McIntosh L (1992). Salicylic acid regulation of respiration in higher plants: alternative oxidase expression. Plant Cell.

[9] Norman C, Howell KA, Millar AH, Whelan JM, Day DA (2004). Salicylic acid is an uncoupler and inhibitor of mitochondrial electron transport. Plant Physiol.

[10] Clifton R, Lister R, Parker KL, Sappl PG, Elhafez D, Millar AH, Day DA, Whelan J (2005). Stress-induced co-expression of alternative respiratory chain components in *Arabidopsis thaliana*. Plant Mol Biol.

[11] Cleland CF, Ajami A (1974). Identification of the flower-inducing factor isolated from aphid honeydew as being salicylic acid. Plant Physiol.

[12] Jin JB, Jin YH, Lee J, Miura K, Yoo CY, Kim WY, Van Oosten M, Hyun Y, Somers DE, Lee I, Yun DJ, Bressan RA, Hasegawa PM (2008). The SUMO E3 ligase, *AtSIZ1*, regulates flowering by controlling a salicylic acid-mediated floral promotion pathway and through affects on *FLC* chromatin structure. Plant J.

[13] Li W, Ahn IP, Ning Y, Park CH, Zeng L, Whitehill JGA, Lu H, Zhao Q, Ding B, Xie Q, Zhou JM, Dai L, Wang GL (2012). The U-box/ARM E3 ligase PUB13 regulates cell death, defense, and flowering time in Arabidopsis. Plant Physiol.

[14] Villajuana-Bonequi M, Elrouby N, Nordström K, Griebel T, Bachmair A, Coupland G (2014). Elevated salicylic acid levels conferred by increased expression of ISOCHORISMATE SYNTHASE 1 contribute to hyperaccumulation of SUMO1 conjugates in the Arabidopsis mutant *early in short days 4*. Plant J.

[15] Fortuna A, Lee J, Ung H, Chin K, Moeder W, Yoshioka K (2015). Crossroads of stress responses, development and flowering regulation--the multiple roles of Cyclic Nucleotide Gated Ion Channel 2. Plant Signal Behav.

[16] Martínez C, Pons E, Prats G, León J (2004). Salicylic acid regulates flowering time and links defence responses and reproductive development. Plant J.

[17] Wang GF, Seabolt S, Hamdoun S, Ng G, Park J, Lu H (2011). Multiple roles of WIN3 in regulating disease resistance, cell death, and flowering time in Arabidopsis. Plant Physiol.

[18] Liu L, Zhang J, Adrian J, Gissot L, Coupland G, Yu D, Turck F (2014). Elevated levels of *MYB30* in the phloem accelerate flowering in *Arabidopsis* through the regulation of FLOWERING LOCUS T. PLoS One.

[19] Lee J, Nam J, Park HC, Na G, Miura K, Jin JB, Yoo CY, Baek D, Kim DH, Jeong JC, Kim D, Lee SY, Salt DE, Mengiste T, Gong Q, Ma S, Bohnert HJ, Kwak SS, Bressan RA, Hasegawa PM, Yun DJ (2006). Salicylic acid-mediated innate immunity in Arabidopsis is regulated by SIZ1 SUMO E3 ligase. Plant J.

[20] Tsuchiya T, Eulgem T (2010). The Arabidopsis defense component EDM2 affects the floral transition in an FLC-dependent manner. Plant J.

[21] Singh V, Roy S, Giri MK, Chaturvedi R, Chowdhury Z, Shah J, Nandi AK (2013). *Arabidopsis thaliana FLOWERING LOCUS D* is required for systemic acquired resistance. Mol Plant Microbe Interact.

[22] Banday ZZ, Nandi AK (2015). Interconnection between flowering time control and activation of systemic acquired resistance. Front Plant Sci.

[23] Thomma BPHJ, Nürnberger T, Joosten MHAJ (2011). Of PAMPS and effectors: the blurred PTI-ETI dichotomy. Plant Cell.

[24] Spoel SH, Dong X (2012). How do plants achieve immunity? Defence without specialized immune cells. Nat Rev Immunol.

[25] Asai S, Shirasu K (2015). Plant cells under siege: plant immune system versus pathogen effectors. Curr Opin Plant Biol.

[26] Seyfferth C, Tsuda K (2014). Salicylic acid signal transduction: the initiation of biosynthesis, perception and transcriptional reprogramming. Front Plant Sci.

[27] Stael S, Kmiecik P, Willems P, Van Der Kelen K, Coll NS, Teige M, Van Breusegem F (2015). Plant innate immunity--sunny side up?. Trends Plant Sci.

[28] Mishina TE, Zeier J (2007). Pathogen-associated molecular pattern recognition rather than development of tissue necrosis contributes to bacterial induction of systemic acquired resistance in Arabidopsis. Plant J.

[29] White RF (1979). Acetylsalicylic acid (aspirin) induces resistance to tobacco mosaic virus in tobacco. Virol.

[30] Malamy J, Carr JP, Klessig DF, Raskin I (1990). Salicylic acid: a likely endogenous signal in the resistance response of tobacco to viral infection. Science.

[31] Métraux JP, Signer H, Ryals J, Ward E, Wyss-Benz M, Gaudin J, Raschdorf K, Schmid E, Blum W, Inverardi B (1990). Increase in salicylic acid at the onset of systemic acquired resistance in cucumber. Science.

[32] Vernooij B, Friedrich L, Morse A, Reist R, Kolditz-Jawhar R, Ward E, Uknes S, Kessmann H, Ryals J (1994). Salicylic acid is not the translocated signal responsible for inducing systemic acquired resistance but is required in signal transduction. Plant Cell.

[33] Pallas JA, Paiva NL, Lamb C, Dixon RA (1996). Tobacco plants epigenetically suppressed in phenylalanine ammonia-lyase expression do not develop systemic acquired resistance in response to infection by tobacco mosaic virus. Plant J.

[34] Brading PA, Hammond-Kosack KE, Parr A, Jones JDG (2000). Salicylic acid is not required for *Cf-2*- and *Cf-9*-dependent resistance of tomato to *Cladosporium fulvum*. Plant J.

[35] Yang Y, Qi M, Mei C (2004). Endogenous salicylic acid protects rice plants from oxidative damage caused by aging as well as biotic and abiotic stress. Plant J.

[36] Manosalva PM, Park SW, Forouhar F, Tong L, Fry WE, Klessig DF (2010). *Methyl Esterase 1* (*StMES1*) is required for systemic acquired resistance in potato. Mol Plant Microbe Interact.

[37] Sánchez G, Gerhardt N, Siciliano F, Vojnov A, Malcuit I, Marano MR (2010). Salicylic acid is involved in the *Nb*-mediated defense responses to *Potato virus X* in *Solanum tuberosum*. Mol Plant Microbe Interact.

[38] Robert-Seilaniantz A, Grant M, Jones JDG (2011). Hormone crosstalk in plant disease and defense: more than just JASMONATE-SALICYLATE antagonism. Annu Rev Phytopathol.

[39] De Vleesschauwer D, Xu J, Höfte M (2014). Making sense of hormone-mediated defense networking: from rice to Arabidopsis. Front Plant Sci.

[40] Dempsey DA, Vlot AC, Wildermuth MC, Klessig DF (2011). Salicylic acid biosynthesis and metabolism. Arabidopsis Book.

[41] Gao QM, Zhu S, Kachroo P, Kachroo A (2015). Signal regulators of systemic acquired resistance. Front Plant Sci.

[42] Kahn MIR, Fatma M, Per TS, Anjum NA, Kahn NA (2015). Salicylic acid-induced abiotic stress tolerance and underlying mechanisms in plants. Front Plant Sci.

[43] Wildermuth MC, Dewdney J, Wu G, Ausubel FM (2001). Isochorismate synthase is required to synthesize salicylic acid for plant defence. Nature.

[44] Strawn MA, Marr SK, Inoue K, Inada N, Zubieta C, Wildermuth MC (2007). *Arabidopsis* Isochorismate synthase functional in pathogen-induced salicylate biosynthesis exhibits properties consistent with a role in diverse stress responses. J Biol Chem.

[45] Nawrath C, Métraux JP (1999). Salicylic acid induction-deficient mutants of Arabidopsis express *PR-2* and *PR-5* and accumulate high levels of camalexin after pathogen inoculation. Plant Cell.

[46] Dewdney J, Reuber TL, Wildermuth MC, Devoto A, Cui J, Stutius LM, Drummond EP, Ausubel FM (2000). Three unique mutants of *Arabidopsis* identify *eds* loci required for limiting growth of a biotrophic fungal pathogen. Plant J.

[47] Uppalapati SR, Ishiga Y, Wangdi T, Kunkel BN, Anand A, Mysore KS, Bender CL (2007). The phytotoxin coronatine contributes to pathogen fitness and is required for suppression of salicylic acid accumulation in tomato inoculated with *Pseudomonas syringae* pv. *tomato* DC3000. Mol Plant Microbe Interact.

[48] Catinot J, Buchala A, Abou-Mansour E, Métraux JP (2008). Salicylic acid production in response to biotic and abiotic stress depends on isochorismate in *Nicotiana benthamiana*. FEBS Lett.

[49] Djamei A, Schipper K, Rabe F, Ghosh A, Vincon V, Kahnt J, Osorio S, Tohge T, Fernie AR, Feussner I, Feussner K, Meinicke P, Stierhof YD, Schwarz H, Macek B, Mann M, Kahmann R (2011). Metabolic priming by a secreted fungal effector. Nature.

[50] Liu T, Song T, Zhang X, Yuan H, Su L, Li W, Xu J, Liu S, Chen L, Chen T, Zhang M, Gu L, Zhang B, Dou D (2014). Unconventionally secreted effectors of two filamentous pathogens target plant salicylate biosynthesis. Nat Com.

[51] Garcion C, Lohmann A, Lamodière E, Catinot J, Buchala A, Doermann P, Métraux JP (2008). Characterization and biological function of the *ISOCHORISMATE SYNTHASE2* gene of Arabidopsis. Plant Physiol.

[52] Huang J, Gu M, Lai Z, Fan B, Shi K, Zhou YH, Yu JQ, Chen Z (2010). Functional analysis of the Arabidopsis *PAL* gene family in plant growth, development, and response to environmental stress. Plant Physiol.

[53] Kim DS, Hwang BK (2014). An important role of the pepper phenylalanine ammonia-lyase gene (*PAL1*) in salicylic acid-dependent signalling of the defence response to microbial pathogens. J Exp Bot.

[54] Bhattacharjee S, Halane MK, Kim SH, Gassmann W (2011). Pathogen effectors target *Arabidopsis* EDS1 and alter its interactions with immune regulators. Science.

[55] Heidrich K, Wirthmueller L, Tasset C, Pouzet C, Deslandes L, Parker JE (2011). *Arabidopsis* EDS1 connects pathogen effector recognition to cell compartment-specific immune responses. Science.

[56] Feys BJ, Moisan LJ, Newman MA, Parker JE (2001). Direct interaction between the *Arabidopsis* disease resistance signaling proteins, EDS1 and PAD4. EMBO J.

[57] García AV, Blanvillain-Baufumé S, Huibers RP, Wiermer M, Li G, Gobbato E, Rietz S, Parker JE (2010). Balanced nuclear and cytoplasmic activities of EDS1 are required for a complete plant innate immune response. PLoS Pathog.

[58] Fu ZQ, Dong X (2013). Systemic acquired resistance: turning local infection into global defense. Annu Rev Plant Biol.

[59] Zhang Y, Xu S, Ding P, Wang D, Cheng YT, He J, Gao M, Xu F, Li Y, Zhu Z, Li X, Zhang Y (2010). Control of salicylic acid synthesis and systemic acquired resistance by two members of a plant-specific family of transcription factors. Proc Natl Acad Sci U S A.

[60] Wang L, Tsuda K, Truman W, Sato M, Nguyen LV, Katagiri F, Glazebrook J (2011). CBP60g and SARD1 play partially redundant critical roles in salicylic acid signaling. Plant J.

[61] van Verk MC, Bol JF, Linthorst HJM (2011). WRKY transcription factors involved in activation of SA biosynthesis genes. BMC Plant Biol.

[62] Wang X, Gao J, Zhu Z, Dong X, Wang X, Ren G, Zhou X, Kuai B (2015). TCP transcription factors are critical for the coordinated regulation of *ISOCHORISMATE SYNTHASE 1* expression in *Arabidopsis thaliana*. Plant J.

[63] Zheng XY, Zhou M, Yoo H, Pruneda-Paz JL, Spivey NW, Kay SA, Dong X (2015). Spatial and temporal regulation of biosynthesis of the plant immune signal salicylic acid. Proc Natl Acad Sci U S A.

[64] Lopez JA, Sun Y, Blair PB, Mukhtar MS (2015). TCP three-way handshake: linking developmental processes with plant immunity. Trends Plant Sci.

[65] Wang L, Tsuda K, Sato M, Cohen JD, Katagiri F, Glazebrook J (2009). Arabidopsis CaM binding protein CBP60g contributes to MAMP-induced SA accumulation and is involved in disease resistance against *Pseudomonas syrinage*. PLoS Pathog.

[66] Gao X, Chen X, Lin W, Chen S, Lu D, Niu Y, Li L, Cheng C, McCormack M, Sheen J, Shan L, He P (2013). Bifurcation of *Arabidopsis* NLR immune signaling via Ca^2+^-dependent protein kinases. PLoS Pathog.

[67] Nomura H, Komori T, Uemura S, Kanda Y, Shimotani K, Nakai K, Furuichi T, Takebayashi K, Sugimoto T, Sano S, Suwastika N, Fukusaki E, Yoshioka H, Nakahira Y, Shiina T (2012). Chloroplast-mediated activation of plant immune signaling in *Arabidopsis*. Nat Commun.

[68] Serrano M, Wang B, Aryal B, Garcion C, Abou-Mansour E, Heck S, Geisler M, Mauch F, Nawrath C, Métraux JP (2013). Export of salicylic acid from the chloroplast requires the multidrug and toxin extrusion-like transporter EDS5. Plant Physiol.

[69] Yamasaki K, Motomura Y, Yagi Y, Nomura H, Kikuchi S, Nakai M, Shiina T (2013). Chloroplast envelope localization of EDS5, an essential factor for salicylic acid biosynthesis in *Arabidopsis thaliana*. Plant Signal Behav.

[70] Heil M, Baldwin IT (2002). Fitness costs of induced resistance: emerging experimental support for a slippery concept. Trends Plant Sci.

[71] Park SW, Kaimoyo E, Kumar D, Mosher S, Klessig DF (2007). Methyl salicylate is a critical mobile signal for plant systemic acquired resistance. Science.

[72] Bartsch M, Bednarek P, Vivancos PD, Schneider B, von Roepenack-Lahaye E, Foyer CH, Kombrink E, Scheel D, Parker JE (2010). Accumulation of isochorismate-derived 2,3-dihydroxybenzoic *3-O-β-*D-xyloside in *Arabidopsis* resistance to pathogens and ageing of leaves. J Biol Chem.

[73] Zhang K, Halitschke R, Yin C, Liu CJ, Gan SS (2013). Salicylic acid 3-hydroxylase regulates *Arabidopsis* leaf longevity by mediating salicylic acid catabolism. Proc Natl Acad Sci U S A.

[74] Zeilmaker T, Ludwig NR, Elberse J, Seidl MF, Berke L, Van Doorn A, Schuurink RC, Snel B, Van den Ackerveken G (2015). DOWNY MILDEW RESISTANT 6 and DMR6-LIKE OXYGENASE 1 are partially redundant but distinct suppressors of immunity in Arabidopsis. Plant J.

[75] Chen Y, Shen H, Wang M, Li Q, He Z (2013). Salicyloyl-aspartate synthesized by the acetyl-amido synthetase GH3.5 is a potential activator of plant immunity in *Arabidopsis*. Acta Biochim Biophys Sin.

[76] Okrent RA, Brooks MD, Wildermuth MC (2009). Arabidopsis GH3.12 (PBS3) conjugates amino acids to 4-substituted benzoates and is inhibited by salicylate. J Biol Chem.

[77] Klessig DF, Tian M, Choi HW (2016). Multiple targets of salicylic acid and its derivatives in plants and animals. Front Immunol.

[78] Malamy J, Klessig DF (1992). Salicylic acid and plant disease resistance. Plant J.

[79] Hayat Q, Hayat S, Irfan M, Ahmad A (2010). Effect of exogenous salicylic acid under changing environment: a review. Environ Exp Bot.

[80] Rivas-San Vicente M, Plasencia J (2011). Salicylic acid beyond defence: its role in plant growth and development. J Exp Bot.

[81] Miura K, Tada Y (2014). Regulation of water, salinity, and cold stress responses by salicylic acid. Front Plant Sci.

[82] Fu ZQ, Yan S, Saleh A, Wang W, Ruble J, Oka N, Mohan R, Spoel SH, Tada Y, Zheng N, Dong X (2012). NPR3 and NPR4 are receptors for the immune signal salicylic acid in plants. Nature.

[83] Wu Y, Zhang D, Chu JY, Boyle P, Wang Y, Brindle ID, De Luca V, Després C (2012). The *Arabidopsis* NPR1 protein is a receptor for the plant defense hormone salicylic acid. Cell Rep.

[84] Manohar M, Tian M, Moreau M, Park SW, Choi HW, Fei Z, Friso G, Asif M, Manosalva P, von Dahl CC, Shi K, Ma S, Dinesh-Kumar SP, O’Doherty I, Schroeder FC, van Wijk KJ, Klessig DF (2015). Identification of multiple salicylic acid-binding proteins using two high throughput screens. Front Plant Sci.

[85] Kumar D (2014). Salicylic acid signaling in disease resistance. Plant Sci.

[86] Kuai X, MacLeod BJ, Després C (2015). Integrating data on the *Arabidopsis* NPR1/NPR3/NPR4 salicylic acid receptors; a differentiating argument. Front Plant Sci.

[87] Mou Z, Fan W, Dong X (2003). Inducers of plant systemic acquired resistance regulate NPR1 function through redox changes. Cell.

[88] Pajerowska-Mukhtar KM, Emerine DK, Mukhtar MS (2013). Tell me more: roles of NPRs in plant immunity. Trends Plant Sci.

[89] Wang D, Amornsiripanitch N, Dong X (2006). A genomic approach to identify regulatory nodes in the transcriptional network of systemic acquired resistance in plants. PLoS Pathog.

[90] Yan S, Dong X (2014). Perception of the plant immune signal salicylic acid. Curr Opin Plant Biol.

[91] Spoel SH, Mou Z, Tada Y, Spivey NW, Genschik P, Dong X (2009). Proteasome-mediated turnover of the transcription coactivator NPR1 plays dual roles in regulating plant immunity. Cell.

[92] Schreinemachers P, Tipraqsa P (2012). Agricultural pesticides and land use intensification in high, middle and low income countries. Food Policy.

[93] Lucas JA, Hawkins NJ, Fraaije BA (2015). The evolution of fungicide resistance. Adv Appl Microbiol.

[94] da Rocha AB, Hammerschmidt R (2005). History and perspectives on the use of disease resistance inducers in horticultural crops. Horttechnology.

[95] Conrath U, Beckers GJM, Langenbach CJG, Jaskiewicz MR (2015). Priming for enhanced defense. Annu Rev Phytopathol.

[96] Gozzo F, Faoro F (2013). Systemic acquired resistance (50 years after discovery): moving from the lab to the field. J Agric Food Chem.

[97] Walters DR, Ratsep J, Havis ND (2013). Controlling crop diseases using induced resistance: challenges for the future. J Exp Bot.

[98] Yoshioka K, Nakashita H, Klessig DF, Yamaguchi I (2001). Probenazole induces systemic acquired resistance in *Arabidopsis* with a novel type of action. Plant J.

[99] Vallad GE, Goodman RM (2004). Systemic acquired resistance and induced systemic resistance in conventional agriculture. Crop Sci.

[100] Conrath U, Beckers GJM, Flors V, García-Agustín P, Jakab G, Mauch F, Newman MA, Pieterse CMJ, Poinssot B, Pozo MJ, Pugin A, Schaffrath U, Ton J, Wendehenne D, Zimmerli L, Mauch-Mani B (2006). Priming: getting ready for battle. Mol Plant Microbe Interact.

[101] Choi HW, Hwang BK (2011). Systemic acquired resistance of pepper to microbial pathogens. J Phytopathol.

[102] van Hulten M, Pelser M, van Loon LC, Pieterse CMJ, Ton J (2006). Costs and benefits of priming for defense in *Arabidopsis*. Proc Natl Acad Sci U S A.

[103] Vane JR (1971). Inhibition of prostaglandin synthesis as a mechanism of action for aspirin-like drugs. Nat New Biol.

[104] Choi HW, Tian M, Song F, Venereau E, Preti A, Park SW, Hamilton K, Swapna GVT, Manohar M, Moreau M, Agresti A, Gorzanelli A, De Marchis F, Wang H, Antonyak M, Micikas RJ, Gentile DR, Cerione RA, Schroeder FC, Montelione GT, Bianchi ME, Klessig DF (2015). Aspirin’s active metabolite salicylic acid targets high mobility group box 1 to modulate inflammatory responses. Mol Med.

[105] Choi HW, Tian M, Manohar M, Harraz MM, Park SW, Schroeder FC, Snyder SH, Klessig DF (2015). Human GAPDH is a target of aspirin’s primary metabolite salicylic acid and its derivatives. PLoS One.

[106] Thomas JO, Travers AA (2001). HMGB1 and 2, and related ‘architectural’ DNA-binding proteins. Trends Biochem Sci.

[107] Hara MR, Thomas B, Cascio MB, Bae BI, Hester LD, Dawson VL, Dawson TM, Sawa A, Snyder SH (2006). Neuroprotection by pharmacologic blockade of the GAPDH death cascade. Proc Natl Acad Sci U S A.

[108] Tian M, Sasvari Z, Gonzalez PA, Friso G, Rowland E, Liu XM, van Wijk KJ, Nagy PD, Klessig DF (2015). Salicylic acid inhibits the replication of *Tomato bushy stunt virus* by directly targeting a host component in the replication complex. Mol Plant Microbe Interact.

[109] Choi HW, Manohar M, Manosalva P, Tian M, Moreau M, Klessig DF. Activation of plant innate immunity by extracellular high mobility group box 3 and its inhibition by salicylic acid. PLoS Pathog. 12:e1005518. doi:10.1371/journal.ppat.1005518. 10.1371/journal.ppat.1005518 PMC4805298 27007252

[110] Paterson JR, Baxter G, Dreyer JS, Halket JM, Flynn R, Lawrence JR (2008). Salicylic acid sans aspirin in animals and man: persistence in fasting and biosynthesis from benzoic acid. J Agric Food Chem.

[111] Lawrence JR, Peter R, Baxter GJ, Robson J, Graham AB, Paterson JR (2003). Urinary excretion of salicyluric and salicylic acids by non-vegetarians, vegetarians, and patients taking low dose aspirin. J Clin Pathol.

[112] Görlach J, Volrath S, Knauf-Beiter G, Hengy G, Beckhove U, Kogel KH, Oostendorp M, Staub T, Ward E, Kessmann H, Ryals J (1996). Benzothiadiazole, a novel class of inducers of systemic acquired resistance, activates gene expression and disease resistance in wheat. Plant Cell.

